# Expression of Myoepithelial Markers in Mammary Carcinomas of 119 Pet Rabbits

**DOI:** 10.3390/ani9100740

**Published:** 2019-09-28

**Authors:** Sophie Degner, Heinz-Adolf Schoon, Sebastian Degner, Mathias Baudis, Claudia Schandelmaier, Heike Aupperle-Lellbach, Sandra Schöniger

**Affiliations:** 1 Institute of Veterinary Pathology, University of Leipzig, 04109 Leipzig, Germany; schoon@vetmed.uni-leipzig.de; 2 Private consultant, full stack developer, Leipzig, Germany; me@sebastiandegner.de; 3 Veterinary Practice Ralf Bischoff, 49328 Melle-Markendorf, Germany; mathias.baudis@ta-praxis-bischoff.de; 4 Laboklin GmbH & Co. KG, Laboratory for clinical diagnostics, 97688 Bad Kissingen, Germany; schandelmaier@laboklin.com (C.S.); aupperle@laboklin.com (H.A.-L.); 5 Targos Molecular Pathology GmbH, 34119 Kassel, Germany

**Keywords:** calponin, histopathology, immunohistochemistry, mammary carcinoma, myoepithelial cells, *Oryctolagus cuniculus*, pet rabbit, statistical analysis

## Abstract

**Simple Summary:**

Mammary cancer is a serious health issue in pet rabbits; prognostic factors are unknown. In a normal mammary gland, glandular secretory cells are surrounded by a single continuous layer of myoepithelial cells. In non-invasive mammary carcinomas, tumor cells are delineated by an intact myoepithelial layer, which is gradually lost to invasive carcinomas. The main aim of this study was to determine in rabbit mammary carcinomas (*n* = 119) the expression of myoepithelial markers that have prognostic significance in human cancer. Results show that all cases contained some retained myoepithelial cells. In 93% of the tumors, neoplastic cells expressed the myoepithelial marker calponin. There was a statistically significant association between higher percentages of calponin-containing cancer cells and histological features indicative of a better tumor differentiation, i.e., a lower proliferation of tumor cells, an increased percentage of tubular growth within the tumor, and a lower tumor grade, respectively. These results suggest that rabbit mammary carcinomas develop from progression of non-invasive cancer forms, and that calponin expression in cancer cells likely represents a favorable prognostic factor. The latter hypothesis has to be confirmed in long-term follow-up studies.

**Abstract:**

Most mammary tumors in pet rabbits are carcinomas; prognostic factors are unknown. The aim of this study on rabbit mammary carcinomas was to determine the expression of myoepithelial markers that have a prognostic relevance in human cancers. Mammary carcinomas (*n* = 119) of female or female-spayed pet rabbits were immunostained for cytokeratin AE1/AE3, vimentin, smooth muscle actin (SMA), and calponin; and percentages of non-neoplastic myoepithelial cells (ME cells) and calponin-positive neoplastic cells were determined. Using statistical analysis, data were correlated with the age of the rabbits and histological tumor characteristics. All carcinomas contained retained spindle-shaped ME, while 115 also contained hypertrophic ME (HME). A statistically significant relationship existed between a higher age and an increase in HME. In 111 carcinomas (93%), tumor cells expressed calponin. There was a significant correlation between higher percentages of calponin-positive tumor cells and a lower mitotic count, an increased percentage of tubular growth, and a lower grading score, respectively. Data suggest that pet rabbit mammary carcinomas develop from progression of in situ cancer and that the extent of calponin expression in tumor cells influences their biological behavior. These results provide the basis for a long-term follow-up on the prognostic significance of calponin expression in mammary cancer cells.

## 1. Introduction

In recent years, rabbits have gained increasing popularity as pets [1]. The most frequent tumor in rabbits is uterine adenocarcinoma [2]. The reported incidence of this tumor in 2–3 years old does is 4%, whereas about 80% of 5–6 years old does are affected [2]. There is an increasing recognition of mammary tumors in pet rabbits [3,4,5]. All tumors with reported sex of the patient occurred in female or female-spayed rabbits [3,4,5], and 67–98% of the tumors were carcinomas [3,4,5]. Most commonly observed were different histotypes of (invasive) adenocarcinomas (78–96%), whereas only some adenosquamous carcinomas (9–11%) [3,4], anaplastic carcinomas (3%) [4], and in situ carcinomas (2–5%) [3,5] were reported. In addition, single cases of a rabbit mammary spindle cell carcinoma [4], as well as carcinosarcoma [6], have been published. So far, the only treatment option is surgical excision.

The detailed molecular characterization of tumors is an important prerequisite for the identification of prognostic markers and features that may predict the response to therapy. In addition, it adds to the basic knowledge of comparative pathology and helps to identify animal models for human cancer. Pet rabbits are very suitable for such investigations because of their medium life expectancy of 6–13 years [7]. 

In breast cancer in women, myoepithelial markers are applied to distinguish in situ carcinomas from invasive carcinomas [8,9]. In addition, there is increasing evidence that cancer associated myoepithelial cells (ME cells) influence tumor progression [9]. Recent data on mouse mammary organoids propose that ME cells prevent proliferation and dissemination of tumor cells by forming a dynamic (instead of a static) barrier [10]. It was shown that the inhibitory actions of ME cells were dependent on their degree of coverage, contractility, and motility, as well as the preservation of cellular adhesions between adjacent ME cells [10].

ME cells of a normal rabbit mammary gland show the concurrent expression of pancytokeratin, vimentin, smooth muscle actin (SMA), p63, and calponin [3]. This is in agreement with the immunostaining of mammary gland ME cells in women [8,11], dogs [12,13,14,15], and cats [13,16]. 

So far, only very limited information on the molecular features of rabbit mammary carcinomas is available [3,5]. A study encompassing 14 mammary adenocarcinomas, two adenosquamous carcinomas, and one matrix-producing carcinoma showed that 13 adenocarcinomas, one adenosquamous carcinoma, and the matrix-producing carcinoma contained not only some non-neoplastic ME cells, but also variable numbers of tumor cells with a myoepithelial differentiation [3]. 

The aim of the present study was to examine a larger case series of mammary carcinomas of pet rabbits for the immunohistochemical expression of myoepithelial markers, and to statistically correlate the results with histopathological features of the tumors and signalment of the rabbits. 

## 2. Materials and Methods 

This retrospective study examined archived, formalin-fixed, paraffin-embedded (FFPE) tissue samples of 119 pet rabbit mammary carcinomas for the immunohistochemical expression of cytokeratin AE1/AE3 (CK), vimentin, calponin, and smooth muscle actin (SMA) (Table 1). The pancytokeratin marker AE1/AE3 detects the cytokeratins 1–6, 8, 10, 14–16, and 19 [17]; thus, in the mammary tissue, this antibody labels luminal epithelial cells and ME cells. Vimentin is an intermediate filament of mainly mesenchymal cells, which is also expressed in some types of myoepithelial cells [14,18]. The applied anti-SMA antibody recognizes α-smooth muscle actin, which represents an actin isoform specific for contractile filaments [19,20]. The applied calponin antibody reacts with calponin 1.

The immunohistochemical results were analyzed by image analysis and were correlated with reported signalment and histopathological features of the 119 carcinomas that had been obtained in a previous study [5].

Signalment: The reported age of the pet rabbits with mammary carcinomas was known for 97 animals and ranged between 1.5 and 10 years of age; the mean age was 5.3 years, with a standard deviation (SD) of 1.59 years. The sex was reported for 107 rabbits; of these, 85 (79%) were female, 22 (21%) were female-spayed [5].

### 2.1. Histopathological Features

For each carcinoma, the percentage of the tumor area with a tubular growth pattern was recorded and ranged from 5.00% to 90.00%. Mitotic figures were counted in 10 high power fields (HPFs), which varied between 0 and 32 [5]. The determination of the histologic tumor grade according to Elston and Ellis [21] showed that 56.00% were grade I (grading scores 3–5), 42.00% were grade II (grading scores 6–7), and 2.00% were grade III (grading scores 8–9) [5]. The obtained total grading scores were as follows: score 3: 2.00%; score 4: 13.00%; score 5: 42.00%; score 6: 21.00%; score 7: 21.00%; and score 8: 2.00% [5].

### 2.2. Immunohistochemistry

Serial sections of all tumors (*n* = 119) were immunostained for CK, vimentin, calponin, and smooth muscle actin (SMA).

The peroxidase antiperoxidase (PAP) method with 3.3´-diaminobenzidine tetrahydrochloride (DAB) as chromogen was used [3]. In the negative controls, primary antibodies were replaced by isotype-matched nonbinding antibodies. All immunostained sections contained immunostained histological structures that served as internal positive control, i.e., epithelium of the adjacent normal mammary tissue or overlying skin (CK), and the vascular tunica media (vimentin, calponin, SMA; Figure S1).

Normal mammary parenchyma was contained in 119 cases. The tubuloalveolar structures were lined by an inner layer of CK-positive secretory epithelial cells and an outer layer of myoepithelial cells that stained positive for CK, vimentin, SMA, and calponin.

### 2.3. Image Analysis 

Per tumor and for each antibody, digital images were taken from three representative areas (S Plan Apo 40× objective, Olympus BH2 microscope), characterized by a predominance of epithelial tumor tissue, the presence of very scant tumor stroma without myofibroblasts, and the absence of tissue necrosis. Digital image analysis was used to determine the epithelial tumor area immunostained for each applied marker and the percentages of epithelial cells positive for these markers.

### 2.4. Measurement of the Immunopositive Tumor Area

**Figure 1:**
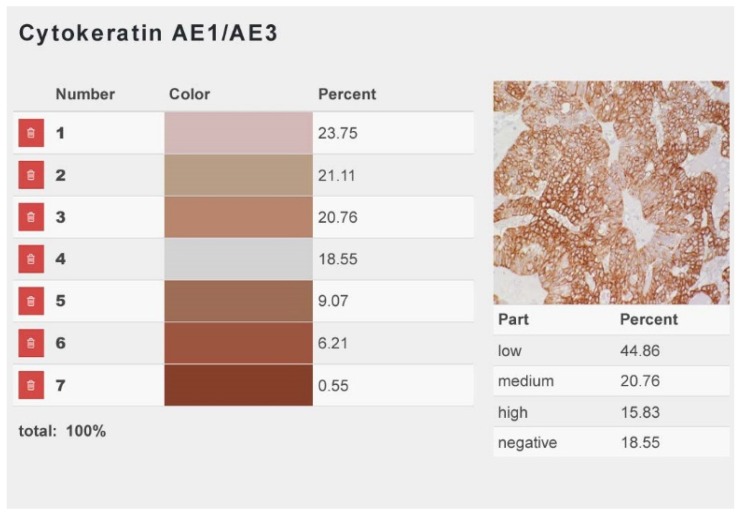
Web application for the detection of the immunopositive areas, specifically designed for the present study. For each uploaded image, the immunopositive areas with low (numbers 1 and 2), moderate (number 3), and high staining intensity (numbers 5, 6, and 7), as well as the immunonegative area (number 4), are calculated. The results are provided as percentages of the entire analyzed area. Exemplarily depicted is an uploaded image from a pet rabbit mammary carcinoma immunostained for cytokeratin AE1/AE3. The total immunostained area encompasses 88.45%. The staining intensity is low in 44.86%, medium in 20.76%, and high in 15.83% of the total immunolabelled area, respectively.

A web application, which uses the script language PHP based on the Laravel framework, was developed specifically for the present study. With this application, a color histogram is created by the initial correction of color gradients and brightness variations and the subsequent determination of the color values by their pixelation. For each color code (low, medium, and high immunostaining intensity), the program automatically provides the immunopositive area as a percent of the entire analyzed area. Per digital image, the total immunopositive area represents the sum of the areas with mild, moderate, and marked immunostaining. From these data, the mean total positive tumor area, as well as the mean tumor areas with mild, moderate, and marked immunostaining, were calculated for each antibody (Figure 1). Measurements were performed on 110 carcinomas. In the other 9 cases, precise measurements of the immunopositive areas were impaired by unspecific staining of secretory material. 

The latter data were used to calculate two immunohistological scores designated as IRS^area^ and H-score^area^ due to their derivation from the IRS (immunoreactive score) [5,22] and H-scores (histological score) [5,23] used for the comparison of cellular immunostaining: IRS^area^ = [(weakly positive area) + (moderately positive area × 5) + (strongly positive area × 10)/100]; H-score^area^ = (weakly positive area) + (moderately positive area × 2) + (strongly positive area × 3). These scores include not only the size of the immunopositive area for a particular marker, but also its staining intensities.

### 2.5. Determination of Immunopositive Cells

For each carcinoma, calponin-, vimentin-, and SMA-positive cells were counted in the three representative digital images (count tool of Adobe Photoshop CS5.1) and divided into three groups (i.e., spindle-shaped non-neoplastic ME cells (SME), hypertrophic non-neoplastic ME cells (HME), and neoplastic cells with a myoepithelial differentiation (NME)). For each stained epithelial cell, its staining intensity (mild, moderate, or marked) was recorded as well. These data were used to calculate the percentages of SME, HME, and NME for each applied marker. 

### 2.6. Statistical Evaluation

The statistical correlation of the immunohistochemical data with the histopathological features of the tumors and the reported signalment was carried out using the R-Studio software (version 1.1.463; R version 3.5.2; R.Studio, Boston, MA). 

To test for a possible linear correlation, regression analysis was performed. A linear model (lm) was implemented by regression analyses and single stratum analysis of variance. Analysis of variance (ANOVA) was used to determine the difference of one or more fitted model objects. Results with *p* < 0.05 were considered as statistically significant. 

The total immunostained areas for CK, calponin, vimentin, and SMA were compared with each other. Similar comparisons for the applied immunohistochemical markers were performed using the IRS^area^ and H-score^area^. In addition, the IRS^area^ and H-score^area^ for each marker were compared with each other. The CK-positive tumor area was correlated with the sex of the pet rabbits. The performed statistical evaluations for the calponin immunostaining are summarized in Table 2.

## 3. Results

### 3.1. Histopathological Features

**Figure 2:**
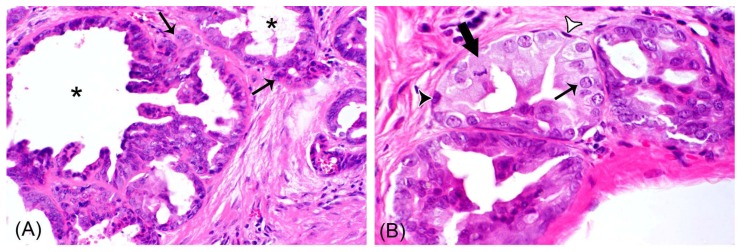
Using histology, all rabbit mammary adenocarcinomas show areas with non-neoplastic myoepithelial cells (ME cells) and areas of invasive carcinoma: (**A**) Depicted is an area composed of neoplastic cells arranged in tubular and cystic structures (asterisks) with multifocal infiltration of the stroma (thin arrows). Hematoxylin–eosin (HE)-stained (**B**) tubular structures are lined by tumor cells (thin arrow) and are surrounded by ME cells. These are spindle-shaped (white arrowhead) or cuboidal to polygonal (black arrowhead). The thick arrow labels a tumor cell with a mitotic figure.

In hematoxylin–eosin-stained tissue section—in addition to epithelial areas entirely composed of tumor cells—all rabbit mammary carcinomas contained nests of tumor cells delineated by spindle-shaped, cuboidal, or polygonal cells, suggestive of retained non-neoplastic ME cells (Figure 2). Epithelial tumor areas and the involved epithelial cell populations were further characterized by immunohistochemistry and image analysis. 

### 3.2. Measurement of the Immunopositive Tumor Area

#### All Rabbit Mammary Carcinomas Contained Epithelial Areas Positive for Vimentin, SMA, and Calponin

CK immunostaining was applied to confirm the immunoreactivity of the tumor tissue, whereas immunostaining for SMA, vimentin, or calponin was used to examine whether the epithelial tumor tissue contained an immunoreactivity for these markers. 

In all examined carcinomas (*n* = 119), the CK-positive epithelial tumor area showed partial immunostaining for SMA, vimentin, or calponin, indicating the presence of epithelial cells immunopositive for one, two, or all three markers.

The CK-positive area was significantly larger than the epithelial tumor areas positive for SMA (*p* = 5.11 × 10^−3^), vimentin (*p* = 1.06 × 10^−5^), and calponin (*p* = 9.17 × 10^−9^). The stained areas for SMA, vimentin, and calponin showed a mild, gradual increase in size (Figure S2). The medians for the positive areas of vimentin (47.95%) and SMA (45.08%) were similar, whereas the median of calponin (52.53%) was higher, and CK showed the highest median value (78.09%) (Table 3 and Figure S2).

Similar results were obtained by consideration not only of the size of the immunopositive area, but also of the obtained staining intensities. The two immunoreactive scores IRS^area^ and H-score^area^ calculated for a particular marker showed a statistically significant correlation (*p* = 2.2 × 10^−16^; *ρ* = 0.94–0.97) for this marker.

The mean IRS^area^ calculated for CK was significantly larger than those data obtained for SMA (*p* = 3.8 × 10^−3^), vimentin (*p* = 2.50 × 10^−4^), and calponin (*p* = 1.58 × 10^−5^). The mean H-score^area^ calculated for CK was significantly larger than the scores obtained for SMA (*p* = 6.30 × 10^−3^,) vimentin (*p* = 1.71 × 10^−4^), and calponin (*p* = 2.31 × 10^−7^). 

The detected smooth muscle actin (SMA)-, vimentin-, or calponin-positive epithelial tumor areas could contain retained non-neoplastic ME cells or tumor cells with the expression of these markers. Thus, as the next step, the morphological features of cells within these areas were further analyzed.

### 3.3. Determination of Immunopositive Cells

#### 3.3.1. Non-Neoplastic Myoepithelial Cells Were Observed in all Mammary Carcinomas

**Figure 3:**
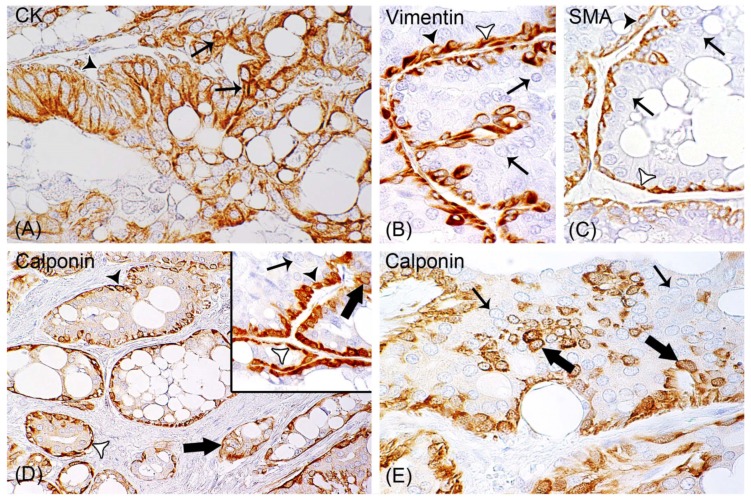
Immunohistochemistry confirming that all rabbit mammary adenocarcinomas contain areas with non-neoplastic myoepithelial cells (ME cells) and areas of invasive carcinoma. In 115/119 (93%) of the tumors, 1.62% to 22% of tumor cells are calponin-positive. (**A**) Tumor cells (thin arrows) and retained ME cells (black arrowhead) are positive for cytokeratin AE1/AE3, with 3.3´-diaminobenzidine tetrahydrochloride (DAB) used as chromogen. (**B**,**C**) Vimentin and smooth muscle actin staining of ME cells that are spindle-shaped (white arrowhead) or cuboidal to polygonal (black arrowhead). Tumor cells are labelled by thin arrows. (**D**) In addition to ME cells (white and black arrowheads), 93% of the carcinomas contain calponin-positive tumor cells (thick arrows). Inset: Depicted in higher magnification are spindle-shaped (black arrowhead) and hypertrophic (white arrowhead) ME cells, calponin-positive tumor cells (thick arrow), and remaining tumor cells (thin arrow). (**E**) Calponin-positive tumor cells (thick arrows) are mixed with calponin-negative carcinoma cells (thin arrows).

As a single layer, non-neoplastic ME cells partially to completely delineated aggregates of carcinomas cells arranged in tubular or cystic structures, papillary projections, or solid aggregates. Non-neoplastic ME cells were solely spindle-shaped in 4/119 (3.40%) tumors, and spindle-shaped and cuboidal to polygonal in 115/119 carcinomas (96.60%) (Figure 3). 

Tumors containing spindle-shaped and cuboidal to polygonal ME cells showed considerable variation in the relative percentages of these two morphological variants. The median values of the spindle-shaped and hypertrophic non-neoplastic ME cells for SMA and vimentin were similar. In contrast to these markers, calponin had a higher median value for spindle-shaped ME cells and a lower median value for hypertrophic ME cells (Table 4 and Figure S3). 

No statistically significant differences existed between the percentages of spindle-shaped ME cells immunopositive for SMA, vimentin, or calponin, as well as between the percentages of cuboidal to polygonal ME cells with SMA, vimentin, or calponin immunostaining (Figure S3). 

With increasing age of the rabbits, mammary carcinomas contained higher percentages of calponin-positive cuboidal to polygonal ME cells (*p* = 0.039; *ρ* = 0.139) and lower percentages of spindle-shaped ME cells (*p* = 0.049; *ρ* = 0.172) (Table 2 and Figure S4).

The subdivision of pet rabbits of this study into two age categories (i.e., those with an age ≤5 years (*n* = 56) and those >5 years of age (*n* = 41)) showed a trend of mammary carcinomas of rabbits >5 years containing higher percentages of hypertrophic calponin-positive ME cells (*p* = 0.060) and lower percentages of spindle-shaped, calponin-positive ME cells (*p* = 0.063) than those of the younger age group.

#### 3.3.2. Calponin-Positive Tumor Cells Were Detected in 93% (111/119) of Carcinomas

NME encompassed 1.62–22.00% (Mean ± SD: 8.00% ± 4.59%) of the total carcinoma cells. They were observed in tumor areas with delineation by non-neoplastic ME cells, and also in invasive tumor areas. NME were ovoid to polygonal and showed moderate anisocytosis and anisokaryosis. They were intermingled as single cells or small aggregates with calponin-negative carcinoma cells, or formed small aggregates surrounded by tumor stroma (Figure 3). 

Since the tumor differentiation is influenced by the percentage of tubular growth, as well as the mitotic count [21], the rabbit mammary adenocarcinomas were examined for a possible association between each of these two parameters and the percentage of calponin-positive tumor cells. The tumor area with a tubular growth varied from 5.00–90.00%, and the number of mitotic figures in 10 HPFs ranged from 0–32. 

**Figure 4:**
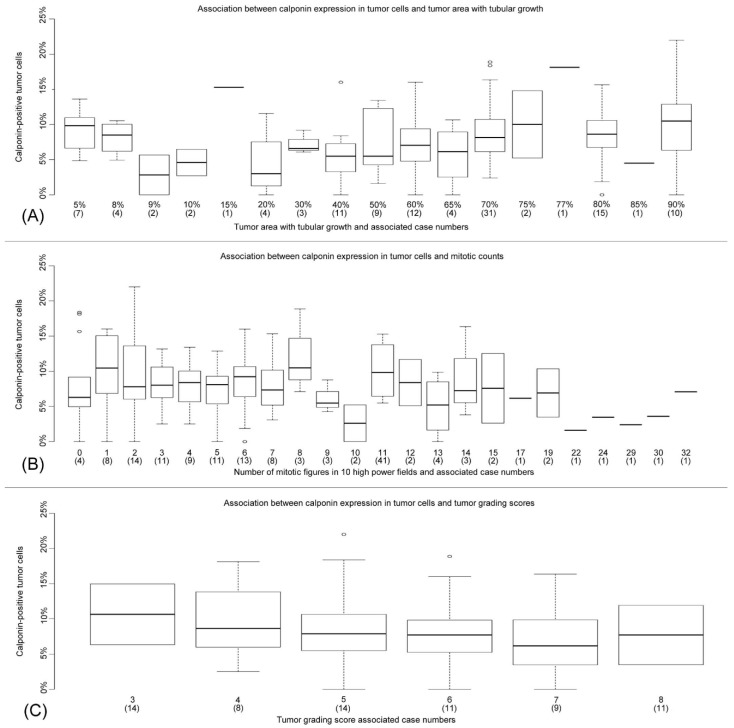
This figure shows the association between the percentages of calponin-positive tumor cells and the tumor area with tubular growth (**A**), the number of mitotic figures in 10 high power fields (**B**), and the tumor grading score (**C**). The numbers in parentheses indicate the number of cases for each category of tubular growth, the different mitotic counts, and the individual grading scores, respectively.

There was a positive correlation between a higher percentage of calponin-positive tumor cells and an increasing percentage of tubular growth (*p* = 0.044; *ρ* = 0.214), as well as a lower number of mitotic figures in 10 HPFs (*p* = 0.026; *ρ* = −0.147), respectively (Figure 4 and Figure S5).

These results strongly indicate that well-differentiated tumors have higher numbers of calponin-positive tumor cells than poorly differentiated tumors. Thus, subsequently the association between the degree of tumor differentiation and the percentages of calponin-positive cells was investigated.

A decrease in the percentages of calponin-positive neoplastic cells was associated with an increase in mitoses per 10 HPFs (*p* = 0.026; *ρ* = −0.147, Figure 4 and Figure S5) and a higher grading score (*p* = 0.048; *ρ* = −0.157; Figure 4). In summary, the better a tumor is differentiated, the higher the number of calponin-positive cells, and vice versa.

## 4. Discussion

### 4.1. Calponin and its Cellular Functions

Three isoforms designated as calponin 1, 2, and 3 exist [24]. Calponin 1 is also referred to as calponin h1 or basic calponin [24,25], and calponin 2 as calponin h2 or neutral calponin [24,25]. In addition to its expression in smooth muscle cells, calponin 1 can be detected in ME cells, myofibroblasts, and cancer cells [24,25]. Calponin 2 is present in smooth muscle, cardiac muscle, and multiple other cell types, including fibroblasts, endothelial cells, and cancer cells [24,25,26]. The distribution of calponin 3 is restricted to neurons, glial cells, and B-lymphocytes [24]. 

Calponin isoforms stabilize actin filaments and modulate cellular motility, proliferation, adhesion, and differentiation [24,25].

The intact layer of non-neoplastic ME cells that surrounds tubuloalveolar structures of the mammary glands has tumor suppressive functions (i.e., it inhibits proliferation and invasion of luminal epithelial cells and maintains their polarity); in addition, it suppresses angiogenesis [11]. ME cells exert their influence on luminal epithelial cells by different mechanisms (i.e., by paracrine signalling and barrier formation) [10,11]. Notably, recent studies revealed dynamic aspects of the ME barrier. In this regard, ME cells not only restrain luminal epithelial cell dissemination, but can even recapture individualized invading cancer cells [10].

In cancer cells, calponin 1 and 2 act as tumor suppressors [24,25]. Their reduced expression facilitates proliferation and migration of cancer cells, as well as their reduced substrate adhesion [24,26]. 

### 4.2. Differences in the Size of the Tumor Areas Stained for CK, Calponin, SMA, and Vimentin

The statistically significant differences in the size of the immunopositive areas between CK and the myoepithelial markers (calponin, SMA, vimentin) were attributed to the immunoreactivity of CK with all epithelial cells (tumor cells and ME cells), whereas calponin stained ME cells and some tumor cells, while vimentin and SMA solely labelled ME cells. These differences were also reflected in the calculated immunoreactive scores (IRS^area^, H-score^area^). In addition to the size of the immunoreactive area, these scores also include the intensity of the immunostaining. The size of the immunoreactive area reflects the number of immunopositive cells, whereas the intensity of the immunostaining is an indicator of the amounts of immunoreactive proteins within the cells. 

The deviations in the cellular expression between the applied markers also explain the differences in their medians (immunopositive areas, IRS^area^ and H-score^area^). In this regard, the slightly higher median values for calponin in comparison to the comparable median values for SMA and vimentin were likely related to the calponin expression in some tumor cells and ME cells, whereas SMA and vimentin were restricted to ME cells.

### 4.3. All Rabbit Mammary Carcinomas Contained Non-Neoplastic Myoepithelial Cells 

The detection of non-neoplastic ME cells in all rabbit mammary carcinomas aligns with results of a previous limited investigation that detected retained non-neoplastic ME cells in 8 of 14 rabbit mammary adenocarcinomas by using the myoepithelial markers calponin and p63 [3].

In contrast to the previous preliminary study [3], in the present investigation, retained non-neoplastic ME cells were further categorized according to their morphological features—they were either spindle-shaped or cuboidal to polygonal.

These two morphological ME cell types were also observed in mammary neoplasms of dogs [13,14,15,27], cats [13], and humans [13]. Beha et al. [14] regarded the spindle-shaped cells as resting ME cells, and the cuboidal to polygonal cells as proliferating ME cells, although the authors did not describe their increase in cell number or the presence of mitotic figures. Due to the absence of the latter features in the examined rabbit mammary carcinomas, the authors prefer the term hypertrophic ME cells, which has been also applied by the authors of [27] for these cells. 

The detection of retained non-neoplastic ME cells in all rabbit mammary adenocarcinomas of this study and the majority of these tumors in another study [5] suggests that rabbit mammary carcinomas mainly develop through progression from non-invasive carcinomas (i.e., in situ carcinomas) [28].

In contrast, the absence of retained ME cells in some rabbit mammary adenocarcinomas [5] may be explained by their advanced stage or de novo development.

### 4.4. A Higher Number of Hypertrophic Myoepithelial Cells Was Associated with Increasing Age of the Rabbits 

Cellular hypertrophy may be a response to age-related changes in the mammary tissue. In human breast tissue, there is experimental evidence that age-related factors induce functional changes in ME cells that can, in turn, influence the gene expression pattern of luminal epithelia [29]. 

In a previous study on the same tumor samples as were used in the present investigation, no statistically significant association existed between the tumor grading scores and the age or the sex (female, female-spayed) of the rabbits [5].

### 4.5. Most Carcinomas Contained Calponin-Positive Tumor Cells

This study confirmed the results of a previous investigation on a limited number of pet rabbit mammary carcinomas that showed the presence of small to moderate numbers of calponin-positive tumor cells in 13 of 14 adenocarcinomas, one of two adenosquamous carcinoma, and a matrix-producing carcinoma by immunostaining for calponin and p63 [3]. 

The detection of calponin, SMA, or p63 in tumor cells suggests their (partial) myoepithelial differentiation [30,31]. Positive immunostaining for p63 has to be interpreted carefully, since this marker is also expressed in areas of squamous differentiation [32]. Therefore, it was not used in the present study. 

Notably, in this study, tumor cells with a calponin immunoreaction were indistinguishable from the remaining neoplastic cells on HE-stained sections. Similarly, calponin-positive neoplastic cells were detected in canine, feline, and human mammary carcinomas that displayed morphological features indicative of being composed of only one tumor cell type [13]. 

Interestingly, in human breast cancer, the (partial) myoepithelial differentiation is mainly observed in estrogen receptor-α (ER-α) negative tumors [31]. In pet rabbits, 63% of mammary carcinomas lacked expression of ER-α and progesterone receptor [5], but 93% contained variable numbers of calponin-positive tumor cells. 

### 4.6. A Correlation Exists between an Increase in Calponin-Positive Tumor Cells and Histological Features Associated with Reduced Proliferation and Higher Differentiation/Lower Tumor Grade

Results of this study suggest that in rabbit mammary carcinomas, calponin expression in tumor cells is associated with a lower proliferation of neoplastic cells and a higher degree of tumor differentiation. Thus, the detection of calponin expression in tumor cells could also possibly represent a favorable prognostic factor in rabbit mammary carcinomas. Although this hypothesis has to be confirmed in additional studies with a long-term follow-up, it aligns with study results on canine mammary carcinomas and different human cancer types. 

Compared to those without myoepithelial differentiation, canine mammary carcinomas containing variable numbers of tumor cells with a myoepithelial differentiation (detected by immunostaining for SMA or p63) showed significantly lower Ki-67 proliferation indices, reduced infiltrative growths, lower rates of vascular or lymphatic invasion, and reduced metastatic spread to regional lymph nodes [33].

In several human cancer types, calponin expression in tumor cells has been revealed as a positive prognostic marker [25,26,34,35]. The comparison of primary and metastatic adenocarcinomas revealed a 17-gene signature associated with metastases that included downregulated expression of calponin 1 [34]. In pancreatic ductal adenocarcinoma, a higher level of calponin 2 expression was associated with reduced lymph node metastasis and prolonged postsurgical survival time [35]. 

In prostate cancer cell lines, lower levels of calponin 2 were associated with faster proliferation, increased migration, and decreased substrate adhesion [26]. Moreover, in a fibrosarcoma cell line, the vector-induced increased expression of calponin 1 correlated with decreased motility and increased substrate adhesion [36]. 

The calponin-positive tumor cells may be derived from the neoplastic transformation of retained non-neoplastic myoepithelial cells or may develop through phenotypic plasticity of cancer cells. In breast cancer of women, there is evidence for the latter [30], whereas ME cells rarely undergo malignant transformation [37].

Notably, in breast cancer of women, myoepithelial differentiation has to be distinguished from the basal-like phenotype of breast cancer. The latter represents an aggressive phenotype and has a poor prognosis [38]. Basal-like breast cancer is characterized by the expression of the so called basal cytokeratins 4, 5, 14, or 17 [38,39]. These cytokeratins, however, are not only expressed in ME cells, but also in some luminal epithelial cells of the mammary gland [38]. 

## 5. Conclusions

This study indicates that rabbit mammary carcinomas mainly develop from progression of in situ cancer. Thus, early clinical excision of mammary tumors in pet rabbits will likely hinder carcinoma progression. The additional detection of calponin expressing tumor cells in the vast majority of these tumors is suggestive of partial myoepithelial differentiation. 

The positive association between higher numbers of calponin-positive tumor cells and a reduced mitotic rate, an increased percentage of tubular growth, and a lower grading score leads to the hypothesis that calponin expression in tumor cells of rabbit mammary carcinomas may be a favorable prognostic factor. The latter assumption would need to be confirmed in a long-term follow-up-study.

## Figures and Tables

**Table 1:** Primary Antibodies Used for Immunostaining of 119 Pet Rabbit Mammary Carcinomas.

Antibody	Monoclonal (Mouse)	Clone	Company	Dilution	Antigen Retrieval
Anti-cytokeratin	antihuman	AE1/AE3	Dako *	1:50	95 °C, citrate buffer
Anti-vimentin	antiporcine	V9	Dako *	1:100	None
Anti-calponin	antihuman	SPM 169	Zytomed *	1:200	95 °C, citrate buffer
Anti-SMA	antihuman	1A4	Dako *	1:100	None

Note: SMA, smooth muscle actin; * Dako, Hamburg, Germany; * Zytomed, Berlin, Germany.

**Table 2:** Statistical Evaluations for Calponin Immunostaining of Pet Rabbit Mammary Carcinomas.

Parameter 1	Parameter 2	Cases	Tests	*p*-Value/*ρ*-Value
Age	Calponin-positive SME	*n* = 97	lm, Src	*p* = 0.049/*ρ* = −0.172
	Calponin-positive HME	*n* = 97	lm, Src	*p* = 0.039/*ρ* = 0.139
	Calponin-positive NME	*n* = 97	lm	*p* = 0.710
Age categories ≤5 years (*n* = 56) >5 years (*n* = 41)	Calponin-positive SME	*n* = 97	lm	*p* = 0.063
	Calponin-positive HME	*n* = 97	lm	*p* = 0.060
	Calponin-positive NME	*n* = 97	lm	*p* = 0.842
sex categories f (*n* = 85) fs (*n* = 22)	Calponin-positive SME	*n* = 97	lm	*p* = 0.730
	Calponin-positive HME	*n* = 107	lm	*p* = 0.943
	Calponin-positive NME	*n* = 107	lm	*p* = 0.164
Percentage of tubular growth	Calponin-positive SME	*n* = 119	lm	*p* = 0.366
	Calponin-positive HME	*n* = 115	lm	*p* = 0.723
	Calponin-positive NME	*n* = 111	lm, Src	*p* = 0.044/*ρ* = 0.214
Mitotic figures/ 10 HPFs	Calponin-positive SME	*n* = 119	lm	*p* = 0.605
	Calponin-positive HME	*n* = 115	lm	*p* = 0.185
	Calponin-positive NME	*n* = 111	lm, Src	*p* = 0.026/*ρ* = −0.147
Grading scores 3–9	Calponin-positive SME	*n* = 119	lm	*p* = 0.993
	Calponin-positive HME	*n* = 115	lm	*p* = 0.547
	Calponin-positive NME	*n* = 111	lm, Src	*p* = 0.048/*p* = −0.157

Note: f, female; fs, female-spayed; HPFs, high power fields; CK, cytokeratin AE1/AE3; SMA, smooth muscle actin; SME, spindle-shaped non-neoplastic myoepithelial cells; HME, hypertrophic non-neoplastic myoepithelial cells; NME, neoplastic cells with a myoepithelial differentiation; lm, linear model; Src, Spearman’s rank correlation.

**Table 3:** Measurement of Tumor Areas Positive for Cytokeratin, Smooth Muscle Actin, Vimentin, and Calponin, as well as IRS and H-Scores in 110 Pet Rabbit Mammary Carcinomas.

Immunostaining/ Marker	Epithelial Tumor Area Range (Median Value)	IRS^area^ Range (Median Value)	H-score^area^ Range (Median Value)
Cytokeratin	13.75% to 100.00% (78.09%)	0.28 to 5.35 (2.06)	17.64 to 199.73 (110.44)
Calponin	5.55% to 87.62% (52.53%)	0.06 to 3.09 (0.96)	5.55 to 126.42 (76.20)
Vimentin	2.80% to 84.18% (47.95%)	0.03 to 2.53 (1.07)	2.80 to 114.05 (67.41)
Smooth muscle actin	1.31% to 88.82% (45.08%)	0.01 to 2.64 (0.78)	1.31 to 124.45 (56.93)

Note. IRS, immunoreactive score; H-score, histological score.

**Table 4:** Percentages of Non-Neoplastic Myoepithelial Cells and Tumor Cells Expressing Smooth Muscle Actin, Vimentin, and Calponin in 119 Pet Rabbit Mammary Carcinomas.

Immunostaining/ Marker	Spindle-Shaped ME (*n* = 119) Range (Median Value)	Hypertrophic ME (*n* = 115) Range (Median Value)	Tumor Cells (*n* = 111) Range (Median Value)
Smooth muscle actin	20.64–100.00% (67.17%)	0.00–79.36% (32.83%)	0.00%
Vimentin	19.85–100.00% (67.41%)	0.00–80.15% (32.59%)	0.00%
Calponin	13.07–100.00% (53.22%)	0.00–70.90% (39.01%)	0.00–21.99% (7.42%)

Note: ME, myoepithelial cells.

## Supplementary Materials

The following are available online at https://www.mdpi.com/2076-2615/9/10/740/s1, Figure S1: Negative and positive controls for the immunostaining of rabbit mammary carcinomas for cytokeratin AE1/AE3, smooth muscle actin, vimentin and calponin, Figure S2: Image analysis to determine immunopositive areas for smooth muscle actin, vimentin, calponin and cytokeratin AE1/AE3, Figure S3: Median values for smooth-muscle actin-, vimentin- and calponin-immunostaining of spindle-shaped and hyperplastic non-neoplastic myoepithelial cells, Figure S4: Association between percentages of spindle-shaped and hypertrophic myoepithelial cells and the age of the rabbits, Figure S5: Scatter plots to depict the relationship between calponin-positive tumor cells and the size of the tumor area with a tubular growth or the mitotic count, respectively.

## References

[1] DeMello M., Pręgowski M.P. (2016). Rabbits multiplying like rabbits: The rise in the worldwide popularity of rabbits as pets. Companion Animals in Everyday Life.

[2] Barthold S.W., Griffey S.M., Percy D.H. (2016). Pathology of Laboratory Rodents and Rabbits.

[3] Schöniger S., Horn L.C., Schoon H.A. (2014). Tumors and tumor-like lesions in the mammary gland of 24 pet rabbits: A histomorphological and immunohistochemical characterization. Vet. Pathol..

[4] Baum B., Hewicker-Trautwein M. (2015). Classification and epidemiology of mammary tumours in pet rabbits (*Oryctolagus cuniculus*). J. Comp. Pathol..

[5] Degner S., Schoon H.-A., Laik-Schandelmaier C., Aupperle-Lellbach H., Schöniger S. (2018). Estrogen receptor-α and progesterone receptor expression in mammary proliferative lesions of female pet rabbits. Vet. Pathol..

[6] Shahbazfar A.A., Mohammadpour H., Isfahani H.R.E. (2012). Mammary gland carcinoma in a New Zealand white rabbit (*Oryctolagus cuniculus*). Acta Sci. Vet..

[7] Arrington L.R., Kelly K.C. (1976). Domestic Rabbit Biology and Production.

[8] Dewar R., Fadare O., Gilmore H., Gown A.M. (2011). Best practices in diagnostic immunohistochemistry: Myoepithelial markers in breast pathology. Arch. Pathol. Lab. Med..

[9] Pandey P.R., Saidou J., Watabe K. (2010). Role of myoepithelial cells in breast tumor progression. Front. Biosci..

[10] Sirka O.K., Shamir E.R., Ewald A.J. (2018). Myoepithelial cells are a dynamic barrier to epithelial dissemination. J. Cell Biol..

[11] Adriance M.C., Inman J.L., Petersen O.W., Bissell M.J. (2005). Myoepithelial cells: Good fences make good neighbors. Breast Cancer Res..

[12] Gama A., Alves A., Gartner F., Schmitt F. (2003). p63: A novel myoepithelial cell marker in canine mammary tissues. Vet. Pathol..

[13] De las Mulas J.M., Reymundo C., de los Monteros A.E., Millán Y., Ordás J. (2004). Calponin expression and myoepithelial cell differentiation in canine, feline and human mammary simple carcinomas. Vet. Comp. Oncol..

[14] Beha G., Sarli G., Brunetti B., Sassi F., Ferrara D., Benazzi C. (2012). Morphology of the myoepithelial cell: Immunohistochemical characterization from resting to motile phase. Sci. World J..

[15] Sánchez-Céspedes R., Millán Y., Guil-Luna S., García-Monterde J., Reymundo C., de los Monteros A.E., de las Mulas J.M. (2011). Myoepithelial cell layer integrity in canine mammary carcinoma. J. Comp. Pathol..

[16] Zappulli V., Caliari D., Rasotto R., Ferro S., Castagnaro M., Goldschmidt M. (2013). Proposed classification of the feline ‘‘complex’’ mammary tumors as ductal and intraductal papillary mammary tumors. Vet. Pathol..

[17] Ordóñez N.G. (2013). Broad-spectrum immunohistochemical epithelial markers: A review. Hum. Pathol..

[18] Grandi D., Campanini N., Becchi G., Lazzaretti M. (2000). On the myoepithelium of human salivary glands. An immunocytochemical study. Eur. J. Morphol..

[19] Gugliotta P., Sapino A., Macrí L., Skalli O., Gabbiani G., Bussolati G. (1988). Specific demonstration of myoepithelial cells by anti-alpha smooth muscle actin antibody. J. Histochem. Cytochem..

[20] Yamin R., Morgan K.G. (2012). Deciphering actin cytoskeletal function in the contractile vascular smooth muscle cell. J. Physiol..

[21] Elston C.W., Ellis I.O. (1991). Pathological prognostic factors in breast cancer. I. The value of histological grade in breast cancer: Experience from a large study with long-term follow-up. Histopathology.

[22] Aupperle H., Ozgen S., Schoon H.A., Schoon D., Hoppen H.O., Sieme H., Tannapfel A. (2000). Cyclical endometrial steroid hormone receptor expression and proliferation intensity in the mare. Equine Vet. J..

[23] Ellis I.O., Lee A.H.S., Pinder S.E., Rakha E.A., Fletcher C.D.M. (2013). Tumors of the breast. Diagnostic Histopathology of Tumors.

[24] Liu R., Jin J.P. (2016). Calponin isoforms CNN1, CNN2 and CNN3: Regulators for actin cytoskeleton functions in smooth muscle and non-muscle cells. Gene..

[25] Taniguchi S. (2005). Suppression of cancer phenotypes through a multifunctional actin-binding protein, calponin, that attacks cancer cells and simultaneously protects the host from invasion. Cancer Sci..

[26] Hossain M.M., Wang X., Bergan R.C., Jin J.P. (2014). Diminished expression of h2-calponin in prostate cancer cells promotes cell proliferation, migration and the dependence of cell adhesion on substrate stiffness. FEBS Open Bio.

[27] Los de Monteros A.E., Millán M.Y., Ordás J., Carrasco L., Reymundo C., las de Mulas J.M. (2002). Immunolocalization of the smooth muscle-specific protein calponin in complex and mixed tumors of the mammary gland of the dog: Assessment of the morphogenetic role of the myoepithelium. Vet. Pathol..

[28] Polyak K., Hu M. (2005). Do myoepithelial cells hold the key for breast tumor progression?. J. Mammary Gland Biol. Neopl..

[29] Miyano M., Sayaman R.W., Stoiber M.H., Lin C.H., Stampfer M.R., Brown J.B., LaBarge M.A. (2017). Age-related gene expression in luminal epithelial cells is driven by a microenvironment made from myoepithelial cells. Aging (Albany NY).

[30] Petersen O.W., Nielsen H.L., Gudjonsson T., Villadsen R., Rønnov-Jessen L., Bissell M.J. (2001). The plasticity of human breast carcinoma cells is more than epithelial to mesenchymal conversion. Breast Cancer Res..

[31] Kesse-Adu R., Shousha S. (2004). Myoepithelial markers are expressed in at least 29% of oestrogen receptor negative invasive breast carcinoma. Mod. Pathol..

[32] Kaufmann O., Fietze E., Mengs J., Dietel M. (2001). Value of p63 and cytokeratin 5/6 as immunohistochemical markers for the differential diagnosis of poorly differentiated and undifferentiated carcinomas. Am. J. Clin. Pathol..

[33] Yoshimura H., Nakahira R., Kishimoto T.E., Michishita M., Ohkusu-Tsukada K., Takahashi K. (2014). Differences in indicators of malignancy between luminal epithelial cell type and myoepithelial cell type of simple solid carcinoma in the canine mammary gland. Vet. Pathol..

[34] Ramaswamy S., Ross K.N., Lander E.S., Golub T.R. (2003). A molecular signature of metastasis in primary solid tumors. Nat. Genet..

[35] Qiu Z., Chu Y., Xu B., Wang Q., Jiang M., Li X., Wang G., Yu P., Liu G., Wang H. (2017). Increased expression of calponin 2 is a positive prognostic factor in pancreatic ductal adenocarcinoma. Oncotarget.

[36] Takeoka M., Ehara T., Sagara J., Hashimoto S., Taniguchi S. (2002). Calponin h1 induced a flattened morphology and supressed the growth of human fibrosarcoma HT1080 cells. Eur. J. Cancer..

[37] Lakhani S.R., O’Hare M.J. (2001). The mammary myoepithelial cell—Cinderella or ugly sister?. Breast Cancer Res..

[38] Rakha E.A., Reis-Filho J.S., Ellis I.O. (2008). Basal-like breast cancer: A critical review. J. Clin. Oncol..

[39] Lakhani S.R., Ellis I.O., Schnitt S.J., Tan P.H., van de Vijver M.J. (2012). WHO Classification of Tumors of the Breast.

