# Supplementary material for: Expression of Myoepithelial Markers in Mammary Carcinomas of 119 Pet Rabbits
Source: Animals (Basel). 2019 Sep 28;9(10):740. doi: 10.3390/ani9100740 (PMC6826665; doi:10.3390/ani9100740)
Supplement: Supplementary file 1 [file animals-09-00740-s001.pdf]

Supplementary materials

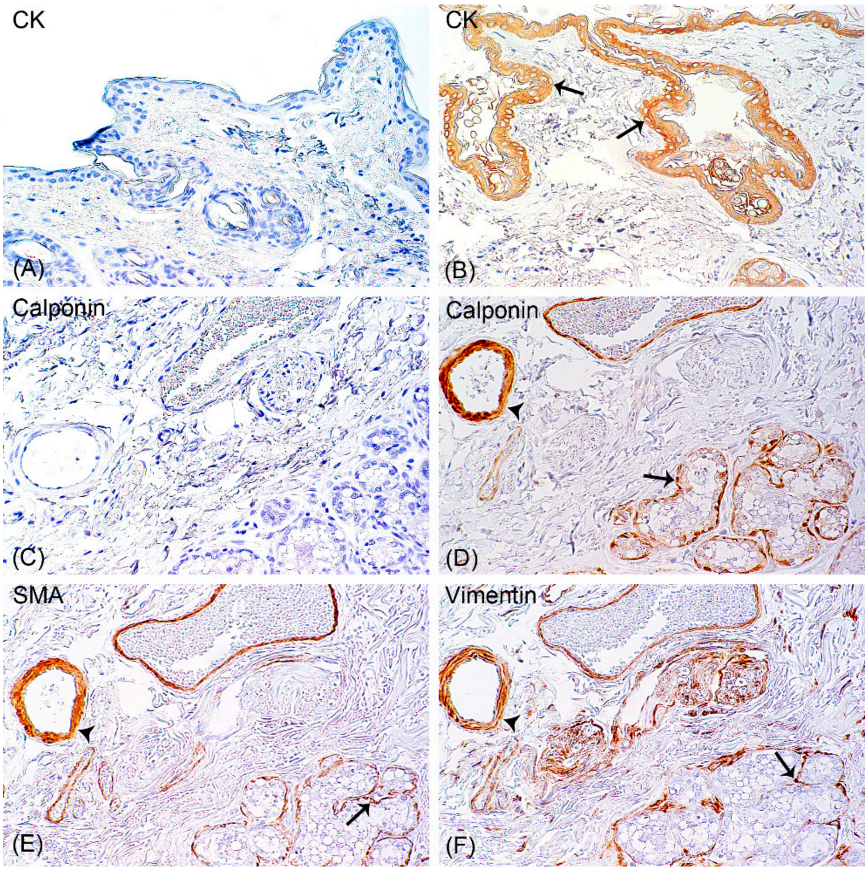

Figure S1. Negative and positive controls for the immunostaining of rabbit mammary carcinomas for cytokeratin AE1/AE3 (CK), smooth muscle actin (SMA), vimentin and calponin. In the negative controls, primary antibodies were replaced by isotype matched nonbinding antibodies. As internal positive controls served the overlying skin (CK), adjacent non-neoplastic mammary tissues (CK, SMA, vimentin, calponin) as well as the tunica media of blood vessels (vimentin, SMA, calponin). In the negative controls there was a complete absence of immunostaining for CK **(A)** and calponin **(C)** as well as vimentin and SMA (data not shown). As internal control, keratinocytes of the epidermis (thin arrows, **B)** and epithelial and myoepithelial cells (ME cells) of the adjacent non-neoplastic mammary tissue (data not depicted) showed a marked cytoplasmic immunolabelling for CK. The internal controls for calponin, SMA and vimentin were the vascular tunica media (arrowhead, **D–F**) and ME-cells of the non-neoplastic mammary tissue (arrows, **D–F)**.

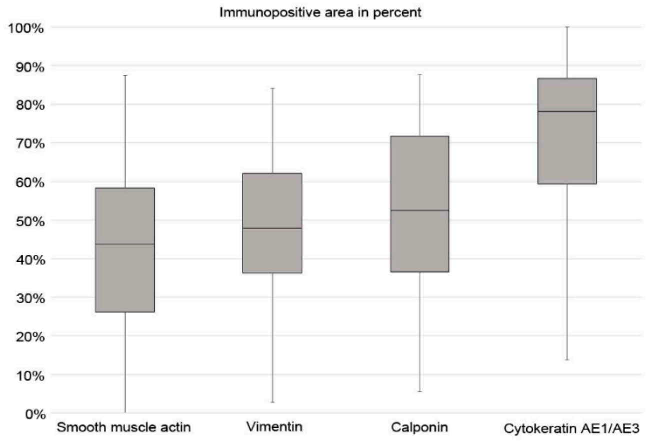

Figure S2. Image analysis to determine immunopositive areas for smooth muscle actin (SMA), vimentin, calponin and cytokeratin AE1/AE3 (CK). Immunopositive areas are calculated in percent of the entire analysed area. The CK-positive area is significantly larger than the epithelial tumor areas positive for SMA, vimentin and calponin. The medians for the positive areas of vimentin (47.95%) and SMA (45.08%) are similar, whereas the median of calponin (52.53%) is higher and CK shows the highest median value (78.09%). These results can likely be explained by the findings of the cellular immunostaining that shows an expression of SMA and vimentin solely in retained non-neoplastic myoepithelial cells (ME cells), whereas calponin is expressed in non-neoplastic ME cells and some tumor cells and CK stains ME cells and all tumor cells.

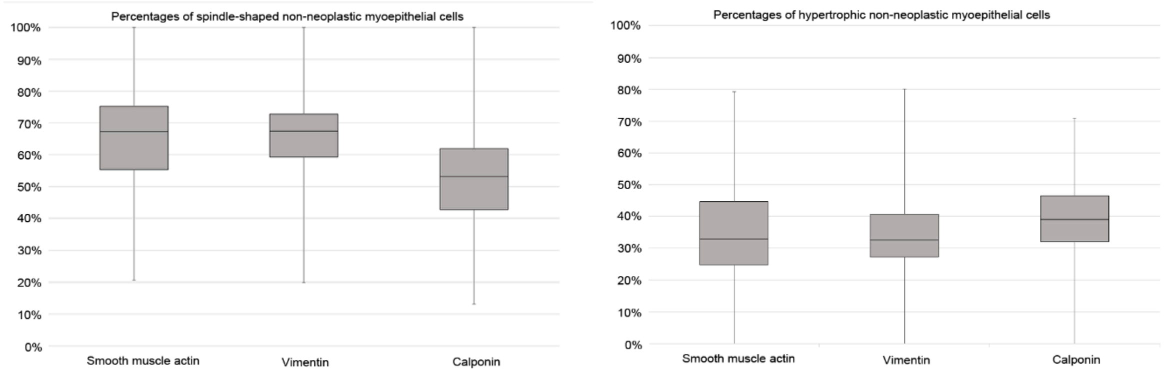

Figure S3. Median values for smooth-muscle actin-, vimentin- and calponin-immunostaining of spindle-shaped and hyperplastic non-neoplastic myoepithelial cells. **(A)** The median values for the spindle-shaped non-neoplastic myoepithelial cells for SMA (67.17%) and vimentin (67.41%) are similar, whereas calponin (53.22%) has a lower median value. **(B)** The median values for the hypertrophic non-neoplastic ME cells for SMA (32.83%) and vimentin (32.59%) are also similar, whereas calponin (39.01%) has a higher median value.

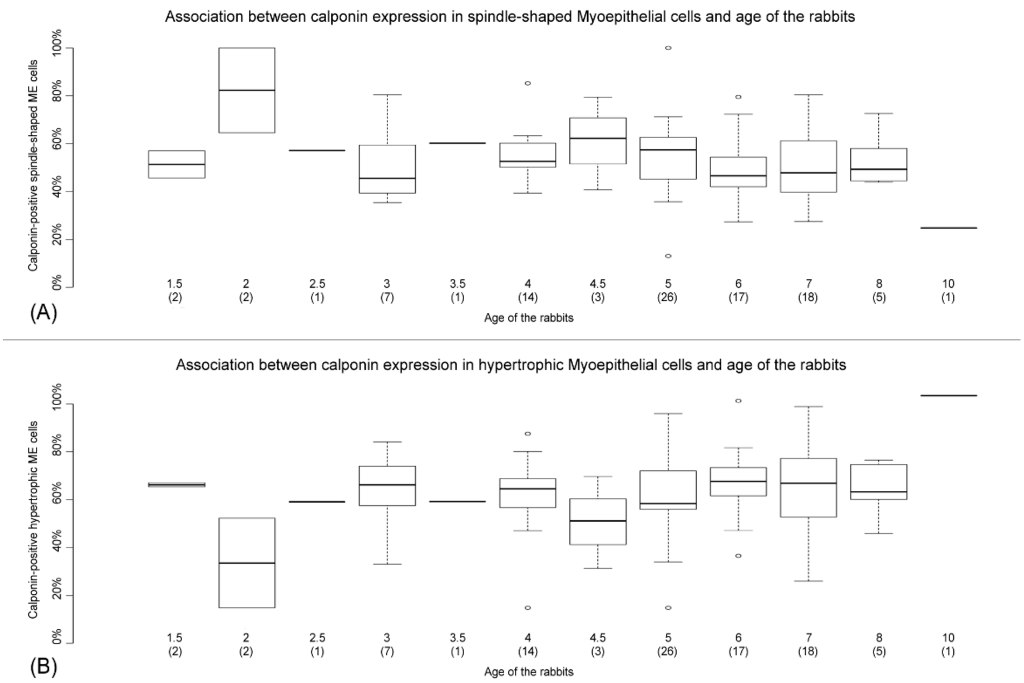

Figure S4. Association between the percentages of spindle-shaped and hypertrophic myoepithelial (ME) cells and the age of the rabbits (n = 97). With an increasing age of the rabbits, mammary carcinomas contained statistically significant lower percentages of spindle-shaped ME cells ($p = 0.049$; $\rho = 0.172$) and higher percentages of calponin-positive cuboidal to polygonal ME cells ($p = 0.039$; $\rho = 0.139$).

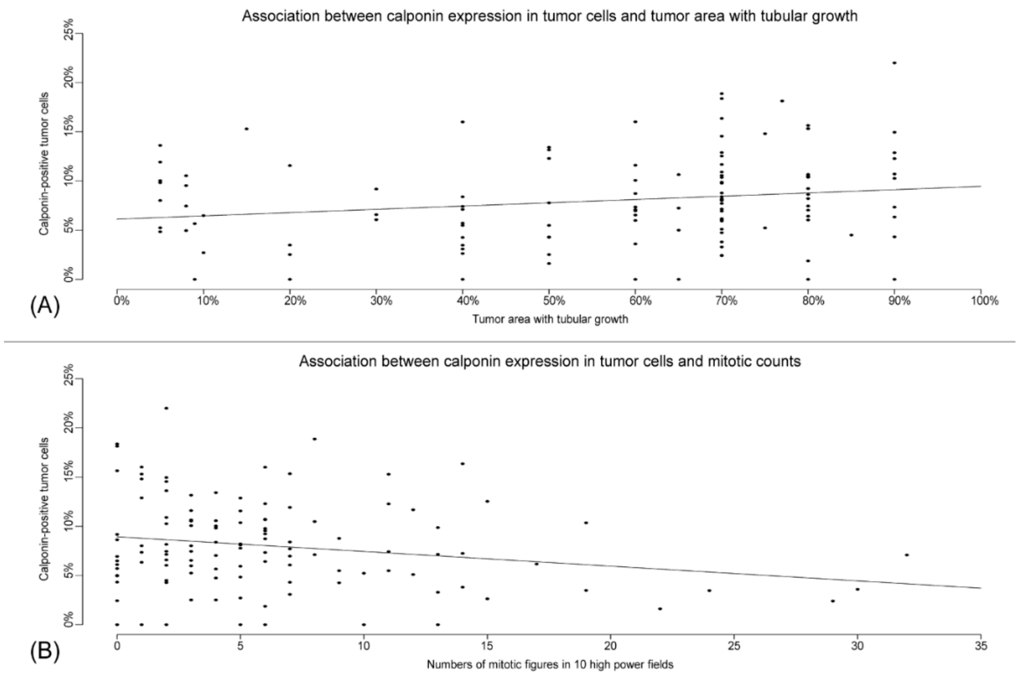

Figure S5. Scatter plots to depict the relationship between calponin-positive tumor cells and the size of the tumor area with a tubular growth **(A)** or the mitotic count **(B)**, respectively. By regression analysis, a statistically significant positive correlation was detected in **(A)** and a negative correlation in (**B).** These data indicate that higher numbers of calponin-positive tumor cells are associated with histological parameters indicative of a better tumor differentiation, i.e. a higher percentage of tubular growth and a lower mitotic count.
